# Flow Cytometric Analysis of T, B, and NK Cells Antigens in Patients with Mycosis Fungoides

**DOI:** 10.1155/2015/856340

**Published:** 2015-12-16

**Authors:** Serkan Yazıcı, Emel Bülbül Başkan, Ferah Budak, Barbaros Oral, Şaduman Balaban Adim, Zübeyde Ceylan Kalin, Güven Özkaya, Kenan Aydoğan, Hayriye Saricaoğlu, Şükran Tunali

**Affiliations:** ^1^Uludag University School of Medicine, Department of Dermatology and Venereology, Bursa, Turkey; ^2^Uludag University School of Medicine, Department of Immunology, Bursa, Turkey; ^3^Uludag University School of Medicine, Department of Pathology, Bursa, Turkey; ^4^Uludag University School of Medicine, Department of Biostatistics, Bursa, Turkey

## Abstract

We retrospectively analyzed the clinicopathological correlation and prognostic value of cell surface antigens expressed by peripheral blood mononuclear cells in patients with mycosis fungoides (MF). 121 consecutive MF patients were included in this study. All patients had peripheral blood flow cytometry as part of their first visit. TNMB and histopathological staging of the cases were retrospectively performed in accordance with International Society for Cutaneous Lymphomas/European Organization of Research and Treatment of Cancer (ISCL/EORTC) criteria at the time of flow cytometry sampling. To determine prognostic value of cell surface antigens, cases were divided into two groups as stable and progressive disease. 17 flow cytometric analyses of 17 parapsoriasis (PP) and 11 analyses of 11 benign erythrodermic patients were included as control groups. Fluorescent labeled monoclonal antibodies were used to detect cell surface antigens: T cells (CD3^+^, CD4^+^, CD8^+^, TCR*αβ* ^+^, TCR*γδ* ^+^, CD7^+^, CD4^+^CD7^+^, CD4^+^CD7^−^, and CD71^+^), B cells (HLA-DR^+^, CD19^+^, and HLA-DR^+^CD19^+^), NKT cells (CD3^+^CD16^+^CD56^+^), and NK cells (CD3^−^CD16^+^CD56^+^). The mean value of all cell surface antigens was not statistically significant between parapsoriasis and MF groups. Along with an increase in cases of MF stage statistically significant difference was found between the mean values of cell surface antigens. Flow cytometric analysis of peripheral blood cell surface antigens in patients with mycosis fungoides may contribute to predicting disease stage and progression.

## 1. Introduction

Mycosis fungoides is the most common variant of primary cutaneous T cell lymphomas. Most of MF patients first present with long-standing reactive inflammatory dermatoses such as PP en plaque so-called premycotic eruptions [1]. Flow cytometric analysis of the cell surface antigens, expressed by peripheral blood cells, is widely used in the diagnosis and also management of hematologic malignancies; however, it has not been used routinely in the evaluation of MF patients. The diagnosis of MF is still challenging with current histopathological and molecular techniques especially in early stages [2]. Neoplastic T cells frequently have an altered level of expression of various surface T cell markers compared with normal T cells. These differences are sufficient to distinguish normal T cells from neoplastic T cells in the same population [3]. Peripheral blood flow cytometry can detect aberrant T cell populations even when there is no lymphocytosis or elevated total white blood cell count. Detection of T cell abnormalities by flow cytometry is increasing in use as an effective and a sensitive method in MF patients [4]. The importance of peripheral blood flow cytometry studies is highlighted by the ISCL/EORTC recommendations for diagnosis that include one or more of the following phenotypical abnormalities demonstrated by flow cytometry including peripheral blood CD4^+^/CD8^+^ rate being above 10, abnormal expression of pan-T cell markers including CD2^+^, CD3^+^, CD4^+^ antigens, and CD7 (CD4^+^CD7^−^ ≥ 40%) antigen loss of T cells [5]. Aberrant expression of T cells, and also B and NK cells markers, may be expressed in patients with MF [6, 7].

The aim of this study was to evaluate the prognostic value of cell surface antigens expressed by T, B, and NK cells and correlated the results with the clinicopathological ISCL MF diagnostic score, retrospectively by 4-color flow cytometry.

## 2. Patients and Methods

### 2.1. Patients

A total of 121 flow cytometric analyses of 121 MF patients, followed up in our clinic between January 2000 and August 2013, were enrolled in the study. All patients had peripheral blood flow cytometry as part of their first visit. TNMB and histopathological staging of the cases were performed in accordance with International Society for Cutaneous Lymphomas/European Organization of Research and Treatment of Cancer (ISCL/EORTC) criteria at the time of flow cytometry sampling retrospectively. 17 flow cytometric analyses of 17 PP and 11 analyses of 11 benign erythrodermic patients were included as control groups (Table 1). By the date of each flow sytometric analysis, TNMB and histopathological staging of cases were performed to all patients according to the Bunn-Lambert staging system and ISCL/EORTC criteria were retrieved from the medical records [5]. To determine prognostic value of flow cytometric analysis, according to the course of the follow-up process of the patients, cases were divided into two groups* stable* and* progressive disease* as recommended.* Stable disease* is described as <25% increase to <50% clearance in skin disease from baseline without new tumors (T_3_) in patients with T_1_, T_2_, or T_4_ only skin disease.* Progressive disease* is described as ≥25% increase in skin disease from baseline or new tumors (T_3_) in patients with T_1_, T_2_, or T_4_ only skin disease or loss of response: in those with complete or partial response, increase of skin score of greater than the sum of plus 50% baseline score [8]. The study was approved by the local ethical committee and conducted according to the principles of the Declaration of Helsinki.

### 2.2. Flow Cytometric Analysis

Peripheral blood specimens were collected in EDTA tubes. Blood samples were transported to the flow cytometry laboratory where the specimens were processed and analyzed within 24 hours of receipt. After incubation of whole peripheral blood with monoclonal antibodies for 15 minutes at room temperature in the dark, erythrocytes were lysed with NH4CL for 10 min, followed by two washing steps using phosphate buffered saline solution. The cells were then resuspended and fixed with 1% paraformaldehyde.

Four-color flow cytometry was performed using FACSCanto (BD Biosciences, USA). Four-color direct immunofluorescent staining was performed as described by the manufacturer, using a 7-tube panel (70 × 15 mm). 100 *μ*L (10^5^ cells) blood samples, containing EDTA, were transferred to tubes and the tubes were placed in the Lyse Wash Assistant (LWA) (BD Biosciences, USA). Fluorescent labeled monoclonal antibodies were used to detect cell surface antigens:* T cells* (CD3^+^, CD4^+^, CD8^+^, TCR*αβ* ^+^, TCR*γδ* ^+^, CD7^+^, CD4^+^CD7^+^, CD4^+^CD7^−^, and CD71^+^),* B cells* (HLA-DR^+^, CD19^+^, and HLA-DR^+^CD19^+^),* NKT cells* (CD3^+^CD16^+^CD56^+^), and* NK cells* (CD3^−^CD16^+^CD56^+^). Fluorescein isothiocyanate (FITC), phycoerythrin (PE), Allophycocyanin (APC), and Peridinin-Chlorophyll-Protein-Complex (PerCP) labeled fluorescent monoclonal antibodies (Beckman Coulter, France) were used. Cells were collected and analyzed on a FACSCanto (BD Biosciences, USA) using FACSDiva (BD Biosciences, USA). The tubes were aspirated until dry to maximize cell yield. Lymphocytes were identified in the standard manner based on CD45 expression and side angle light scatter.

### 2.3. Statistical Analysis

SPSS for Windows Version 20.0 (SPSS Inc, Chicago, Illinois, USA) software package program was used for the statistical analysis. Variables were represented with mean, standard deviation, median, and maximum-minimum values. Categorical values of the variables given to cross-tables and differences between the two groups were analysed with Pearson's chi-square and Fisher's exact test. Median and mean values of binary groups compared with using Kruskal-Wallis and Mann-Whitney tests, and for more than two groups ANOVA test was used. ROC curve analysis was performed to determine the cut-off values between progressive and stable disease groups. *P* < 0.05 was considered to be significant.

## 3. Results

A total of 121 MF patients (70 M, 51 F) were included in the study. The age of MF patients during sampling ranged from 18 to 89 years (mean ± SD; 54.42 ± 13.81). The age of 17 parapsoriasis patients (6 M, 11 F) ranged from 17 to 62 (mean ± SD; 46.99 ± 12.05). The age of benign erythroderma patients ranged from 36 to 83. MF patients were followed up for an average of 4.28 ± 2.96 years, 105 cases were accepted as stable, and 16 cases were progressive. Clinical and demographic features of MF patients and histopathological and TNM staging of cases according to the Bunn-Lambert staging system and ISCL/EORTC criteria were summarized in Table 2.

The mean value of all cell surface antigens was not statistically significant between parapsoriasis and MF groups. This finding may show that flow cytometry is not enough alone to differentiate mycosis fungoides from benign dermatoses and parapsoriasis. CD3^+^CD16^+^CD56^+^ *cells* may differentiate benign erythrodermic and MF patients assessed with two-sided *t*-test (*P* = 0.027) (Table 3). According to histopathological and clinical stage in cases of mycosis fungoides, statistically significant differences were found between the mean values of all cell surface antigens (Tables 4 and 5). While increasing stage of disease the number of CD3^+^, CD4^+^, TCR*αβ* ^+^, CD4^+^CD7^+^, CD4^+^CD7^−^, and CD71^+^ cells and CD4^+^/CD8^+^ cell percentage were significantly increased and the number of CD8^+^, CD7^+^, HLA-DR^+^, CD19^+^, HLA-DR^+^, CD19^+^, CD3^−^CD16^+^, and CD56^+^ cells decreased significantly (Table 5). These findings may be useful in identifying advanced stage cases in patients with MF.

When MF patients were examined in two groups as stable and progressive, all cell surface antigens except CD4^+^CD7^−^ cells may play a role in determining the progression. According to ROC curve analysis results, flow cytometric results suggestive of disease progression were as follows: mean CD3^+^ cell percentage >79.1; mean CD4^+^ cell percentage >49.2; mean CD8^+^ cell percentage ≤5.2; CD4^+^/CD8^+^ percentage >2.4; mean TCR*αβ* ^+^ cell percentage >73.1; TCR*γδ* ^+^ cell percentage ≤ 2.4; CD7^+^ cell percentage ≤48; CD4^+^CD7^+^ cell percentage >47.8; CD4^+^CD7^−^ cell percentage >9.4; CD71^+^ cell percentage >3.4; HLA-DR^+^ cell percentage ≤23.1; CD19^+^ cell percentage ≤6; HLA-DR^+^CD19^+^ cell percentage ≤5; CD3^+^CD16^+^CD56^+^ cell percentage ≤1; CD3^−^CD16^+^CD56^+^ cell percentage ≤9.9 in peripheral blood at any time (Table 6).

## 4. Discussion

The diagnosis of MF remains a challenging area in dermatopathology and conclusive diagnosis is often difficult, necessitating the terms “diagnostic of,” “consistent with,” and “suggestive of” to describe the disease and histopathologic finding needs to be confirmed with the immunohistochemical studies with some limitations in between [9]. The cluster of differentiation (termed as Cluster of Designation or Classification Determinant) (CD) is a protocol used for the identification and investigation of cell surface molecules for immunophenotyping of cells. The use of combining markers has allowed more specific definitions for cell types within the immune system than one molecule [10, 11]. Previous studies using flow cytometry to analyze skin biopsy specimens for the diagnosis of CTCL and MF have been performed and reported that flow cytometry is a highly sensitive and specific diagnostic test for MF [4, 12, 13]. In addition to skin biopsy specimens, flow cytometric analysis has proven to be an efficient and sensitive method to detect and enumerate MF/SS cells in the peripheral blood [3, 14, 15]. Depending on the degree of differentiation of neoplastic cells one or more altered expressions of T cell related antigens including CD2^+^, CD3^+^, CD4^+^, CD7^+^, or CD26^+^, were identified in 92% of cases as positive for malignant T cell clones, with loss of CD26^+^ being the most common (70%), and CD56^+^ cases have been reported [16, 17]. However, increased CD26^+^ cells, or CD7^+^ cells, and even altered expression levels of other more specific T cell markers, such as CD3^+^ and CD4^+^, were also observed in nonneoplastic samples. That loss or dim expression of CD7 and CD26 can be found in patients with benign inflammatory dermatoses and aberrant dim CD3 can be observed in nonneoplastic T cells [4]. Flow cytometric studies of the peripheral blood may show expansion of the CD4^+^CD7^−^ population reflective of circulating atypical lymphocytes of Sezary type [18]. Lymphoma cells in the peripheral blood, especially at high levels, have been recognized as an independent adverse prognostic indicator in patients with mycosis fungoides, and the International Society for Cutaneous Lymphomas and the Cutaneous Lymphoma Task Force of European Organization for Research and Treatment of Cancer revised the mycosis fungoides/Sezary syndrome staging criteria in 2007 to incorporate blood (B) involvement as a major prognostic factor and defined the criteria for B-staging. Natural Killer activity of peripheral blood mononuclear cells in patients with MF was investigated with controlled study and revealed that patients with advanced disease had a significant defect of Natural Killer activity [19]. T cell lymphomas with aberrant CD20 (B cell surface antigen) expression, associated with worse prognosis, have been reported [20–22]. Hristov et al. reported that changes in abnormal populations parallel changes in patient's clinical course and increasing numbers of abnormal T cells were associated with worsening disease, and authors suggested that flow cytometry provides valuable information for diagnosis, prognosis, and therapeutic efficacy in MF/SS [23].

To the best of our knowledge this is the largest study to assess the prognostic role and clinical utility of flow cytometry in patients with MF. Peripheral blood samples as a diagnostic and tool for MF. Although, in cases of plaque or tumoral stage of MF, immunophenotypical abnormalities are significant, the significance of flow cytometric analysis in early stage MF is limited. Our findings suggest that T cell phenotype affects the clinical behaviour of MF compatible with the previous reports.

## 5. Conclusion

We concluded that flow cytometric analysis of peripheral blood cell surface antigens in patients with mycosis fungoides may contribute to identifying an advanced stage and predicting the disease progression. Flow cytometry is less time consuming and is prognostic tool for the management of MF.

## Figures and Tables

**Table 1:** Demographic features of the groups included in the study.

	Mycosis fungoides	Parapsoriasis	Benign erythroderma
*n*/flow analysis	121/121	17/17	11/11
Gender			
♀/♂	51/70	11/6	6/5
Flow age (y)			
Mean ± SD	54.42 ± 13.81	46.99 ± 12.05	62.23 ± 15.78
Median; min.–max.	54.0; 18–90	46.00 ± 17–62	68.50; 36–83
Duration of disease (y)			
Mean ± SD	7.14 ± 5.60	2.94 ± 2.46	—
Median; min.–max.	5.0; 1–35	2.0 ± 1–9	1; 1–20

**Table 2:** Clinical and demographic features of MF patients and histopathological and TNM staging of cases according to the Bunn-Lambert staging system and ISCL/EORTC criteria.

*n*	121
Gender	
♀/♂	51/70
Age (y)	
Mean ± SD	54.42 ± 13.81
Median; min.–max.	54.0; 18–90
Duration of disease (y)	
Mean ± SD	7.14 ± 5.60
Median; min.–max.	5.0; 1–35
Follow-up period (y)	
Mean ± SD	4.28 ± 2.96
Median; min.–max.	4; 1–14
Coursing	
Stable	105
Progressive	16
Eksitus	5
Histopathological stage	
Patch	75
Plaque	37
Tumor	9
Clinical (T) stage	
T_1_	33
T_2_	68
T_3_	7
T_4_	13
Lymph node (N) involvement	
None	109
Positive	12
TNM staging	
Early (IA –IIA)	
IA	33
IB	67
IIA	1
Late (IIB–IV)	
IIB	6
III	10
IV	4

**Table 3:** The mean value of all cell surface antigens between groups.

CD molecule	Mycosis fungoides	Parapsoriasis	Benign erythroderma	
	*n*: 121	*n*: 17	*n*: 11	
CD3^+^				0.917
Mean ± SD	73.66 ± 9.62	71.12 ± 3.33	62.97 ± 23.26	
Median; min.–max.	73.60; 48.70–98.70	70.90; 67.70–74.70	58.70; 43.60–90.90	
CD4^+^				0.754
Mean ± SD	47.54 ± 13.26	43.0 ± 8.81	39.62 ± 27.09	
Median; min.–max.	45.3 ± 15.30–97.50	48.70; 31.20–50.40	32.05; 15,80–78.60	
CD8^+^				0.954
Mean ± SD	23.49 ± 10.62	25.96 ± 4.86	20.95 ± 9.86	
Median; min.–max.	22.0; 0.50–73.30	24.9; 21.40–32.60	20.3; 10.60–32.60	
CD4^+^/CD8^+^				0.836
Mean ± SD	5.61 ± 18.69	1.74 ± 0.6	2.40 ± 2.09	
Median; min.–max.	2.0; 0.2–183.2	2.0; 0.90–2.30	1.95; 0.60–5.10	
TCR*αβ* ^+^				0.944
Mean ± SD	68.96 ± 9.66	67.88 ± 4.0	58.80 ± 20.71	
Median; min.–max.	69.10; 45.80–98.0	69.60; 63.10–72.50	53.35; 42.0–86.50	
TCR*γδ* ^+^				0.056
Mean ± SD	4.09 ± 2.91	3.10 ± 2.10	3.35 ± 4.43	
Median; min.–max.	3.20; 0.10–13.60	4.30; 0.80–4.80	1.20; 1.0–10.0	
CD7^+^				0.585
Mean ± SD	74.22 ± 12.60	80.0 ± 3.77	77.4 ± 4.35	
Median; min.–max.	76.60; 2.80–98.0	79.2; 74.60–84.0	79.05; 71.10–80.40	
CD4^+^CD7^+^				0.478
Mean ± SD	38.98 ± 11.50	40.68 ± 7.40	37.22 ± 17.86	
Median; min.–max.	38.60; 9.60–96.50	44.80; 31.10–46.80	31.75; 22.30–63.10	
CD4^+^CD7^−^				0.193
Mean ± SD	9.62 ± 11.53	4.52 ± 1.43	4.95 ± 3.87	
Median; min.–max.	5.60; 0.50–88.90	4.90; 2.20–6.10	3.40; 2.30–10.70	
CD71^+^				0.091
Mean ± SD	5.70 ± 9.98	1.80 ± 0.71	15.65 ± 23.69	
Median; min.–max.	3.0; 0.1–71.0	1.90; 0.8–2.80	4.95; 1.6–51.1	

B cell surface antigens				
HLA-DR^+^				0.368
Mean ± SD	30.47 ± 10.05	28.06 ± 5.81	37.22 ± 16.46	
Median; min.–max.	29.2; 2.20–60.20	26.4; 20.6–36.0	33.6; 21.80–59.90	
CD19^+^				0.468
Mean ± SD	11.66 ± 4.81	12.74 ± 4.77	9.40 ± 3.84	
Median; min.–max.	11.70; 1.0–24.80	10.90; 8.90–20.80	9.95; 4.90–12.80	
HLA-DR^+^CD19^+^				0.559
Mean ± SD	10.80 ± 4.80	12.22 ± 4.70	8.20 ± 3.34	
Median; min.–max.	10.70; 0.30–24.70	10.40; 8.70–20.10	50; 4.60–11.20	

NKT cells surface antigens				
CD3^+^CD16^+^CD56^+^				0.027
Mean ± SD	3.31 ± 3.26	1.54 ± 0.78	1.92 ± 1.30	
Median; min.–max.	2.20; 0.20–20.50	1.20; 0.90–2.80	1.95; 0.70–3.10	

NK cells surface antigens				
CD3^−^CD16^+^CD56^+^				0.523
Mean ± SD	12.92 ± 8.06	13.56 ± 5.28	16.87 ± 1.83	
Median; min.–max.	12.80; 0.10–39.80	13.60; 7.30–21.50	17.55; 2.20–30.20	

**Table 4:** The mean value of all cell surface antigens according to histopathological stage.

Histopathological stage	Patch	Plaque	Tumor	*P* value
*n*	101	50	12	
T cells surface antigens				
CD3^+^				0.000↑
Mean ± SD	71.26 ± 7.72	75.16 ± 9.12	88.90 ± 7.68	
Median; min.–max.	71.50; 51.30–89.10	76.30; 48.70–92.20	88.80; 75.40–98.70	
CD4^+^				0.000↑
Mean ± SD	44.56 ± 8.32	47.22 ± 12.03	77.76 ± 15.58	
Median; min.–max.	44.50; 28.30–73.60	47.60; 20.60–66.20	79.60; 55.50–97.50	
CD8^+^				0.016↓
Mean ± SD	24.24 ± 9.32	25.99 ± 10.52	7.98 ± 8.56	
Median; min.–max.	22.25; 9.40–73.30	24.75; 11.40–66.30	3.80; 0.50–22.50	
CD4^+^/CD8^+^				0.005↑
Mean ± SD	2.18 ± 1.16	2.89 ± 5.23	43.41 ± 55.33	
Median; min.–max.	1.90; 0.20–7.80	1.80; 0.30–35.30	19.20; 2.50–183.20	
TCR*αβ* ^+^				0.000↑
Mean ± SD	66.63 ± 8.14	69.26 ± 8.22	86.39 ± 8.43	
Median; min.–max.	67.70; 46.10–87.10	69.25; 45.80–86.50	85.10; 73.80–98.0	
TCR*γδ* ^+^				0.006↓
Mean ± SD	4.35 ± 2.97	4.26 ± 2.75	1.28 ± 4.43	
Median; min.–max.	3.60; 0.60–13.60	3.35; 0.50–11.20	1.20; 0.10–10.0	
CD7^+^				0.044↓
Mean ± SD	76.68 ± 10.28	71.99 ± 9.32	73.05 ± 27.14	
Median; min.–max.	78.15; 2.80–93.10	72.20; 44.50–87.0	73.60; 11.70–98.0	
CD4^+^CD7^+^				0.003↑
Mean ± SD	38.91 ± 8.48	35.89 ± 8.22	51.34 ± 27.20	
Median; min.–max.	9.65; 13.40–54.50	35.3; 16.0–53.10	48.70; 9.60–96.50	
CD4^+^CD7^−^				0.000↑
Mean ± SD	7.10 ± 6.76	10.17 ± 7.94	27.70 ± 28.06	
Median; min.–max.	5.0; 1.0–51.30	7.40; 2.20–35.90	20.80; 0.50–88.90	
CD71^+^				0.000↑
Mean ± SD	4.47 ± 7.77	3.94 ± 4.66	22.21 ± 21.28	
Median; min.–max.	2.60; 0.10–71.0	2.40; 0.30–23.1	13.20; 3.60–57.70	

B cells surface antigens				
HLA-DR^+^				0.000↓
Mean ± SD	31.10 ± 9.76	32.49 ± 7.39	17.69 ± 12.73	
Median; min.–max.	28.45; 11.40–60.20	31.80; 18.80–50.40	16.30; 2.20–43.70	
CD19^+^				0.020↓
Mean ± SD	11.55 ± 4.74	12.84 ± 4.44	8.11 ± 5.29	
Median; min.–max.	11.40; 1.50–24.80	12.75; 4.70–22.50	10.0; 15.70	
HLA-DR^+^CD19^+^				0.041↓
Mean ± SD	10.79 ± 4.65	11.87 ± 4.36	6.80 ± 5.86	
Median; min.–max.	10.65; 1.20–24.70	11.95; 2.90–20.50	7.80; 0.30–15.50	

NKT cells surface antigens				
CD3^+^CD16^+^CD56^+^				0.000↓
Mean ± SD	3.38 ± 3.46	3.61 ± 3.01	1.60 ± 1.86	
Median; min.–max.	2.10; 0.20–20.50	2.55; 0.50–12.30	0.90; 0.20–6.20	

NK cells surface antigens				
CD3^−^CD16^+^CD56^+^				0.000↓
Mean ± SD	14.82 ± 8.14	11.73 ± 6.30	2.20 ± 2.35	
Median; min.–max.	14.40; 2.60–39.80	11.50; 2.10–35.80	1.50; 0.10–8.0	

↓: decreasing with stage; ↑: increasing with stage.

**Table 5:** The mean values of all cell surface antigens according to clinical stage.

Clinical (T) stage	T_1_	T_2_	T_3_	T_4_	*P* value
*n*	47	95	6	16	
T cells surface antigens					
CD3^+^					0.000↑
Mean ± SD	73.93 ± 7.07	71.31 ± 9.01	88.41 ± 8.67	77.25 ± 17.64	
Median; min.–max.	74.50; 62.30–92.60	71.80; 48.70–96.50	87.70; 79.10–98.70	85.35; 43.60–93.20	
CD4^+^					0.000↑
Mean ± SD	44.63 ± 9.42	44.49 ± 8.60	75.18 ± 19.31	60.27 ± 24.06	
Median; min.–max.	44.70; 15.30–66.20	44.70; 20.60–63.90	74.35; 55.50–97.50	65.20; 15.80–92.30	
CD8^+^					0.005↓
Mean ± SD	26.75 ± 10.56	24.12 ± 9.36	11.41 ± 10.32	14.23 ± 9.68	
Median; min.–max.	23.20; 14.10–73.30	23.45; 9.40–66.30	11.60; 0.50–22.50	15.10; 1.50–32.60	
CD4^+^/CD8^+^					0.000↑
Mean ± SD	2.7 ± 5.16	2.19 ± 1.21	51.10 ± 74.46	13.97 ± 20.21	
Median; min.–max.	1.90; 0.20–35.30	1.80; 0.30–7.80	10.35; 2.50–183.20	3.90; 0.60–61.50	
TCR*αβ* ^+^					0.000↑
Mean ± SD	68.99 ± 7.42	66.68 ± 8.28	86.65 ± 9.48	71.68 ± 17.65	
Median; min.–max.	68.60; 50.0–87.10	68.0; 45.80–86.0	86.3; 76.90–98.0	76.0; 42.0–93.70	
TCR*γδ* ^+^					0.011↓
Mean ± SD	5.19 ± 3.30	3.95 ± 2.63	1.66 ± 0.92	2.35 ± 2.66	
Median; min.–max.	4.40; 0.80–13.60	3.25; 0.50–11.20	2.10; 0.40–2.60	1.45; 0.10–10.0	
CD7^+^					0.001↓
Mean ± SD	77.89 ± 6.33	74.19 ± 11.33	79.63 ± 15.65	61.73 ± 21.63	
Median; min.–max.	78.20; 58.60–93.10	76.10; 2.80–89.30	77.75; 54.70–98.0	66.50; 11.70–83.0	
CD4^+^CD7^+^					0.859↑
Mean ± SD	38.91 ± 8.26	37.25 ± 8.41	64.85 ± 23.52	37.67 ± 17.19	
Median; min.–max.	40.10; 13.40–53.10	37.05; 16.0–53.60	54.30; 42.50–96.50	35.45; 9.60–71.30	
CD4^+^CD7^−^					0.002↑
Mean ± SD	7.71 ± 7.75	7.81 ± 6.40	11.40 ± 16.17	24.02 ± 25.18	
Median; min.–max.	5.70; 1.70–51.30	5.20; 1.0–35.90	4.65; 0.50–42.50	15.75; 2.30–88.90	
CD71^+^					0.002↑
Mean ± SD	3.31 ± 2.78	4.79 ± 8.39	14.36 ± 15.71	17.45 ± 21.59	
Median; min.–max.	2.50; 0.3–11.30	3.0; 0.10–71.0	7.80; 3.60–44.60	6.50; 1.4–57.70	

B cells surface antigens					
HLA-DR^+^					0.001↓
Mean ± SD	29.46 ± 8.79	32.37 ± 9.11	21.56 ± 15.76	28.15 ± 15.30	
Median; min.–max.	27.50; 19.60–56.40	31.10; 11.40–60.20	22.0; 3.80–43.70	25.60; 2.20–59.90	
CD19^+^					0.021↓
Mean ± SD	11.37 ± 3.96	12.48 ± 4.89	8.13 ± 6.41	8.67 ± 4.37	
Median; min.–max.	11.70; 1.50–19.10	11.95; 3.20–24.80	6.80; 1.0–15.70	8.50; 2.30–18.10	
HLA-DR^+^CD19^+^					0.030↓
Mean ± SD	10.53 ± 3.92	11.64 ± 4.79	6.78 ± 7.17	7.72 ± 4.31	
Median; min.–max.	10.60; 1.20–17.80	11.20; 2.90–24.70	4.75 ± 0.30–15.50	6.95; 0.40–17.40	

NKT cells surface antigens					
CD3^+^CD16^+^CD56^+^					0.006↓
Mean ± SD	3.46 ± 3.10	3.57 ± 3.50	2.06 ± 2.12	1.50 ± 1.16	
Median; min.–max.	2.50; 0.30–16.60	2.25; 0.20–20.50	1.60; 0.20–6.20	1.25; 0.20–3.80	

NK cells surface antigens					
CD3^−^CD16^+^CD56^+^					0.000↓
Mean ± SD	13.28 ± 6.34	14.34 ± 8.30	2.90 ± 2.92	8.86 ± 9.84	
Median; min.–max.	13.10; 2.60	13.60; 2.10–39.80	2.70; 0.10–8.0	3.30; 0.10–30.20	

↓: decreasing with stage; ↑: increasing with stage.

**Table 6:** ROC curve analysis of the investigated cell surface antigens to predict the cut-off value.

Cell surface antigen	Cut-off value criterion	Area under curve	*P* value	Sensitivity (95% CI)	Specificity (95% CI)
T cells surface antigens					
CD3^+^	**>79.1**	0.859	0.001	87.50 (61.6–98.1)	84.56 (22.5–55.2)
CD4^+^	**>49.2**	0.880	0.001	87.50 (61.6–98.1)	84.56 (77.7–90.0)
CD8^+^	**≤5.2**	0.835	0.001	62.5 (35.5–84.7)	98.66 (95.2–99.8)
CD4^+^/CD8^+^	**>2.4**	0.873	0.001	93.75 (69.7–99.0)	69.80 (61.70–77.0)
TCR*αβ* ^+^	**>73.1**	0.887	0.001	92.86 (66.1–98.8)	72.29 (71.6–85.7)
TCR*γδ* ^+^	**≤2.4**	0.847	0.001	85.71 (57.2–97.8)	73.38 (65.2–80.5)
CD7^+^	≤48	0.647	0.036	78.57 (49.2–95.1)	50.74 (42.0–59.4)
CD4^+^CD7^+^	>47.8	0.680	0.028	50 (23.1–76.9)	89.51 (83.3–94.0)
CD4^+^CD7^−^	>9.4	0.660	0.053	64.29 (35.2–87.1)	82.27 (74.9–88.2)
CD71^+^	**>3.4**	0.831	0.001	92.86 (66.1–98.8)	63.43 (54.7–71.6)

B cells surface antigens					
HLA-DR^+^	**≤23.1**	0.734	0.001	56.25 (26.9–80.2)	97.3 (93.2–99.2)
CD19^+^	**≤6**	0.691	0.001	43.75 (19.8–70.1)	89.86 (83.8–94.2)
HLA-DR^+^CD19^+^	**≤5**	0.723	0.001	56.25 (29.9–80.2)	85.14 (78.4–90.4)

NKT cells surface antigens					
CD3^+^CD16^+^CD56^+^	≤1	0.649	0.023	68.75 (41.4–88.9)	62.16 (53.8–70.0)

NK cells surface antigens					
CD3^−^CD16^+^CD56^+^	**≤9.9**	0.869	0.001	75.0 (47.6–92.6)	92.52 (87.0–96.2)
